# Knot my problem: Overcoming transradial catheter complications

**DOI:** 10.1002/ccr3.2537

**Published:** 2019-11-12

**Authors:** Melanie Walker, Michael R. Levitt, Basavaraj V. Ghodke

**Affiliations:** ^1^ Department of Neurological Surgery University of Washington School of Medicine Seattle Washington; ^2^ Stroke and Applied Neuroscience Center University of Washington School of Medicine Seattle Washington; ^3^ Departments of Neurological Surgery, Radiology and Mechanical Engineering University of Washington School of Medicine Seattle Washington; ^4^ Departments of Radiology and Neurological Surgery University of Washington School of Medicine Seattle Washington

**Keywords:** catheter, endovascular, knot, radial

## Abstract

Not all complications from transradial access can be prevented, even with diligent patient selection and preprocedure planning. This brief visual report offers technical suggestions to reverse knots and kinks encountered during catheter manipulation for endovascular transradial cerebral procedures.

## Graphical abstract

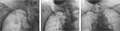

A growing body of evidence supports the adoption of transradial artery access in cerebral interventions due to reduced rates of complications, bleeding, and improved patient satisfaction when compared to transfemoral approaches.^1^ We present simple techniques for managing commonly encountered in challenging situations. Loops (Figure 1A) occur with over‐twisting of the catheter while forming the reverse curve. A single loop will straighten by slowly advancing the glidewire to the tip of the catheter. Tortuous anatomy (Figure 1B) can lead to redundancy or even an outright knot. In the case where there is air (arrow) or the catheter tip is wedged into a vessel origin, first attempt a gentle forward push to dislodge the catheter tip from the ostia and then advance the glidewire slowly toward the tip, directed toward the descending aorta. Do not pull the catheter as it may knot or advance further into the vessel and introduce air. Finally, the lemniscate kink (Figure 1C) does not require traction or glidewire as the tip is already in the vessel of interest, a slight twist will undo the shape. If simple techniques fail, options include encircling the knot with a larger sheath or transfemoral snare capture. We recommend appropriate patient screening if additional imaging is available, use of intra‐arterial anti‐spasm vasodilating medication “cocktail”^2^ upon access, a minimum 5‐French catheter sizes, and hydrophilic glidewires to minimize complications.

**Figure 1:**
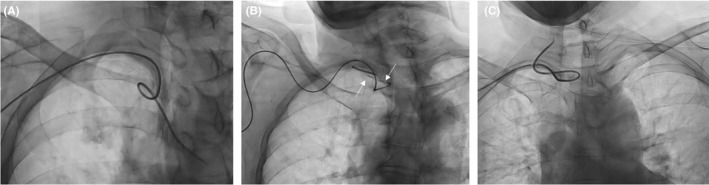
Catheter loops (A), tortuous subclavian artery with trapped air (B, white arrows), and leminscate loops (C) can be managed with simple techniques

## CONFLICT OF INTEREST

None declared.

## AUTHOR CONTRIBUTIONS

All three authors were operators in these cases and assisted in manuscript and image preparation.
